# Liver Transplantation in a Child With Homozygous Familial Hypercholesterolemia: A Case Report and Literature Review

**DOI:** 10.31083/RCM39753

**Published:** 2025-09-24

**Authors:** Chongxia Zhong, Zhu Li, Yihai Liu, Biao Xu, Lina Kang

**Affiliations:** ^1^Department of Cardiology, Nanjing Drum Tower Hospital, Affiliated Hospital of Medical School, Nanjing University, 210008 Nanjing, Jiangsu, China‌

**Keywords:** homozygous familial hypercholesterolemia, liver transplantation, case report

## Abstract

Homozygous familial hypercholesterolemia (HoFH) is a rare inherited metabolic disorder. Meanwhile, HoFH is characterized by extremely high plasma levels of low-density lipoprotein cholesterol (LDL-C) from birth, alongside xanthomas and premature atherosclerotic cardiovascular diseases (ASCVDs). Traditional drugs such as statins have difficulty maintaining serum lipids at an ideal level. Here, we report the case of a 12-year-old child with HoFH who underwent liver transplantation. The goal of lipid reduction could not be achieved in this patient by any other means, and the patient had also experienced mild cardiovascular damage. During the 5-year post-transplant follow-up, the serum lipids were controlled in the patient, while the progression of atherosclerotic plaques was detected without the use of any lipid-lowering drugs. Additionally, we review the progress of current treatments for HoFH and discuss new lipid-lowering medications, as well as the challenges associated with liver transplantation.

## 1. Introduction

Homozygous familial hypercholesterolemia (HoFH) is an inherited lipid metabolism disorder characterized by a significant increase in low-density lipoprotein cholesterol (LDL-C) to >13 mmol/L, accompanied by xanthomas and premature atherosclerotic cardiovascular diseases (ASCVDs) [1]. HoFH is a rare disease, with a prevalence of between 1,000,000 and 160,000. Without treatment, these patients usually die of coronary heart disease before the age of 30 [2].

Low-density lipoprotein receptor (*LDLR*), low-density lipoprotein receptor adaptor protein 1 (*LDLRAP1*), apolipoprotein B (*ApoB*) and proprotein convertase subtilisin kexin-9 (*PCSK9*) are the main pathogenic genes in HoFH [3]. As the major ligand of LDLR, mutation of ApoB leads to the failure of LDL binding to LDLR. The acquired functional mutation of PCSK9 causes excessive degradation of LDLR. LDLRAP1 is an adaptor protein of LDLR, and homozygous mutation of the *LDLRAP1* gene may lead to dysfunction in the liver uptake and delivery of LDL. *LDLR* gene mutations are found in approximately 80% of HoFH patients. These cause impairment or loss of function of LDLR, which binds and clears LDL-C in the blood. The type of mutation in LDLR determines the residual LDL receptor function. For example, receptor negative is defined as a loss-of-function variant, while receptor defective is defined as a milder (hypomorphic) pathogenic change. Accordingly, the therapeutic strategy for HoFH is to control plasma LDL-C under the normal range as soon as possible. Although classical and new lipid-lowering drugs such as statins, ezetimibe and PCSK9 inhibitors have been developed, they cannot effectively regulate plasma lipids to within the target value. The first liver transplantation aimed at curing HoFH was conducted in 1984 [4]. Since then, many successful cases of liver transplantation for HoFH have been reported worldwide, and this approach is considered to be the only effective treatment for curing HoFH.

Here, we report the case of a HoFH child who received a liver transplant and subsequently experienced decreased plasma levels of total cholesterol (TC) and LDL-C. Furthermore, we review the current treatment options for HoFH patients.

## 2. Case Presentation

**Fig. 1.**
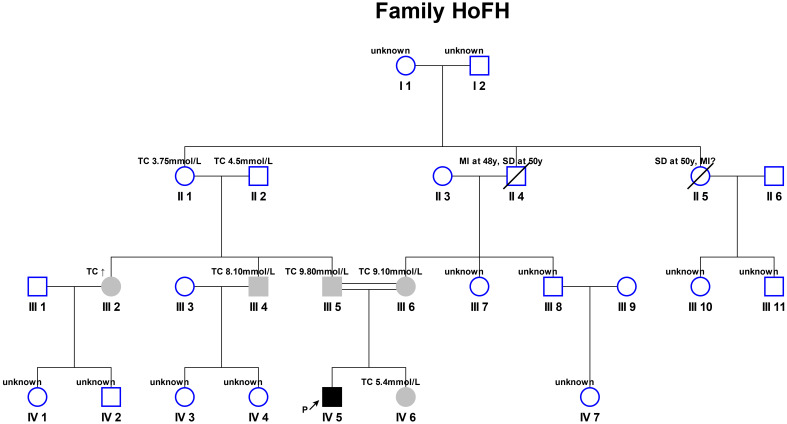
**Pedigree of the patients’ family**. The proband was delivered as the second child of a consanguineous marriage. HoFH, homozygous familial hypercholesterolemia; TC, total cholesterol; MI, myocardial infarction. The ↑ indicates the patient’s reported elevated TC; the slash (╱) indicates patient death; the ↗ indicates a prompt, where “P” stands for proband; circular, female; square, male; black, homozygous; grey, heterozygous; blank, unknown.

A 12-year-old male patient was admitted to our hospital for coronary artery evaluation in preparation for liver transplantation. He first presented 7 years earlier with white, grain-sized nodules in interphalangeal joints. One year later, he displayed progressive swelling in interphalangeal joints, and his plasma level of TC was 21.04 mmol/L (normal reference range: 3–5.7 mmol/L), LDL-C was 19.61 mmol/L (normal reference range: 2.70–3.36 mmol/L), triglycerides (TGs) was 1.39 mmol/L (normal reference range: ≤1.7 mmol/L) and high-density lipoprotein cholesterol (HDL-C) was 0.8 mmol/L (normal reference range: 1.03–1.55 mmol/L). He was the second child of a consanguineous marriage, and his parents and sister also had elevated levels of TC and LDL-C. The plasma TC level of his parents and sister ranged from 7.68 to 10.28 mmol/L, and their plasma LDL-C level ranged from 5.51 to 8.02 mmol/L. He was clinically diagnosed with familial hypercholesterolemia (FH) and treated with simvastatin. However, the levels of TC and LDL-C remained high, and xanthomas appeared on his wrists, knees, elbows, ankles and buttocks. Two years later, the patient experienced morning stiffness in his limbs, the xanthomas progressed to tumor-like nodules, and his TC plasma level was 23.36 mmol/L, LDL-C was 21.17 mmol/L. The patient attended another hospital, where his treatment was adjusted to statin and ezetimibe. Meanwhile, a genetic study identified a homozygous mutation in *LDLR*: exon 4, nucleotide *c.459delC*, amino acid p.Gln154Serfs52^*^. The same gene mutation was also detected in his parents, sister, uncle and grandmother, all of whom were heterozygous for the mutation (Fig. 1). Considering the poor control of plasma lipid levels, it was suggested the patient consider undergoing lipoprotein apheresis (LA).

Four years later, the patient attended the Department of Cardiology at our hospital for an evaluation of cardiovascular status in preparation for liver transplantation. The plasma levels of TC and LDL-C were 22.49 mmol/L and 15.94 mmol/L, respectively. The xanthomas in his eyelids, wrists, knees, elbows, ankles and buttocks had progressed further, and the patient also had mild liver damage from the consumption of statins. Ultrasound examination of the carotid artery indicated an increased intimal thickness (IMT; left: 0.1 cm, right: 0.12 cm) and a flat echogenic plaque (1.39 × 0.18 cm) can be seen on the anterior wall of the left carotid artery trunk. In addition, echocardiography demonstrated mild tricuspid regurgitation, and excess velocity (2.33 m/s) in the descending aortic arch. Moreover, there were mild plaques of the coronary artery left anterior descending branch (LAD) during cardiac catheterization. After taking liver damage into consideration, the lipid-lowering strategy was adjusted to ezetimibe (10 mg, once daily) and PCSK9 (420 mg, once a month). The TC and LDL-C levels subsequently decreased to 15.65 mmol/L and 13.8 mmol/L, respectively.

**Fig. 2.**
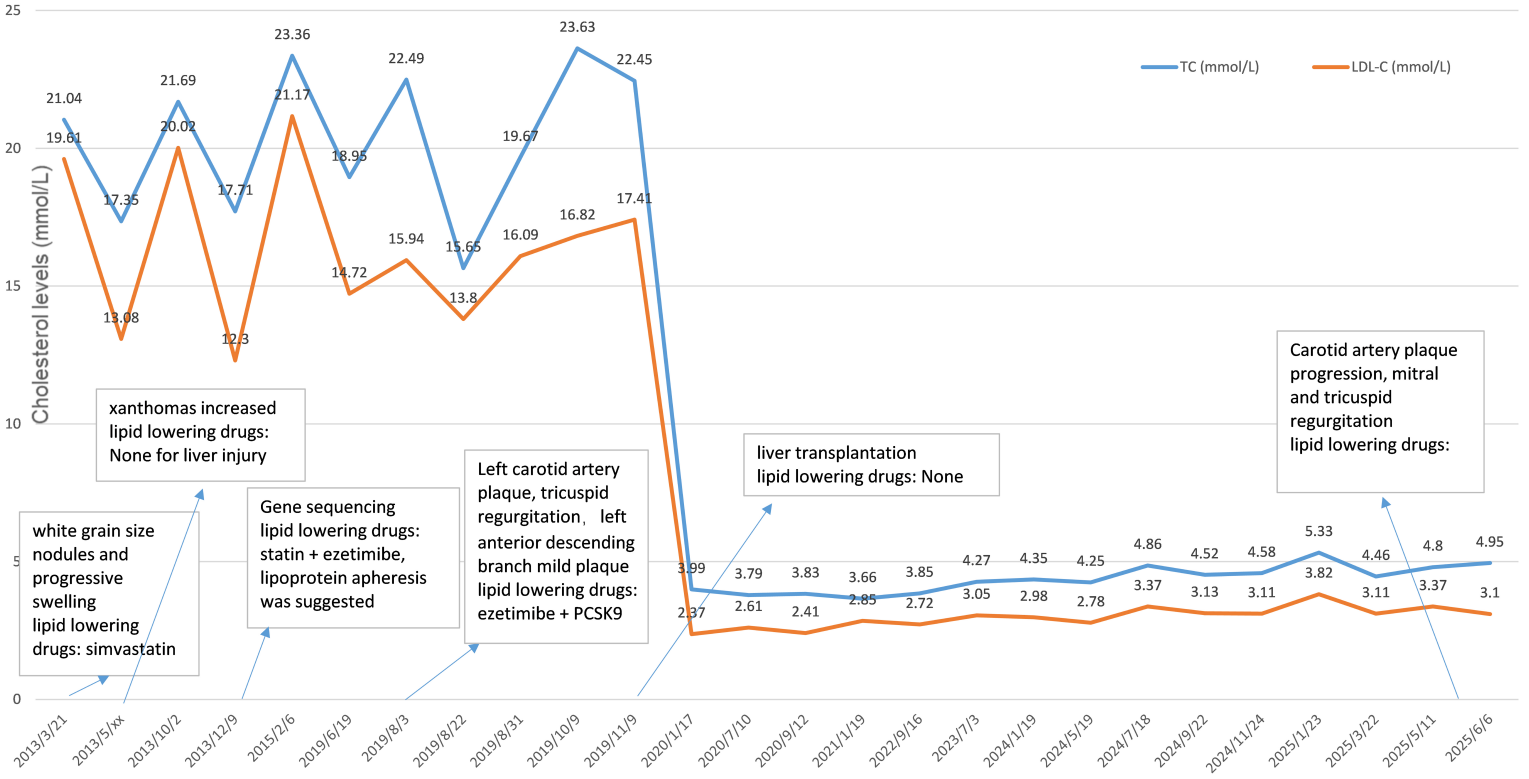
**Changes in the patients’ cholesterol level**. The TC and LDL-C plasma levels decreased rapidly within one month after liver transplantation, and reached the normal range without use of additional lipid-lowering strategies. LDL-C, low-density lipoprotein cholesterol; PCSK9, proprotein convertase subtilisin kexin-9.

**Fig. 3.**
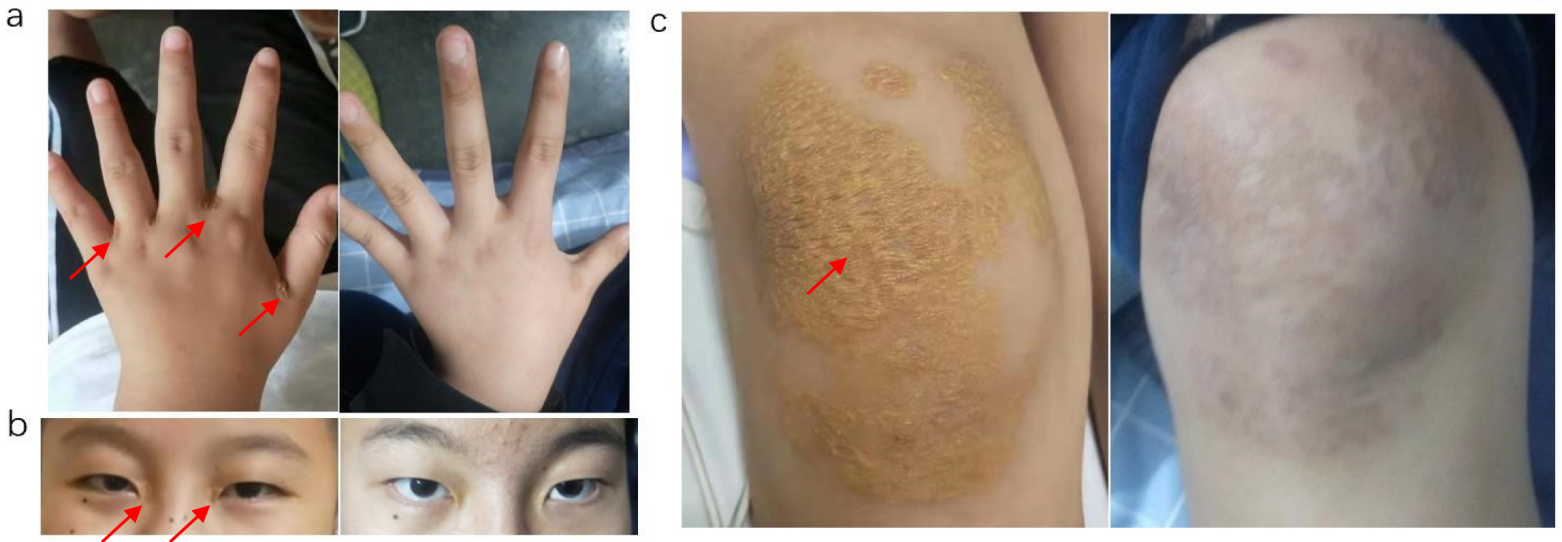
**Comparison of xanthoma before and after operation**. Red arrows indicate xanthoma. Xanthomas initially present in the interphalangeal joints (a), eyelids (b) and knee joints (c) subsequently disappeared after liver transplantation. The left and right photographs of a, b, and c correspond to preoperative and postoperative times, respectively.

Liver transplantation was performed four months after cardiac catheterization, with no intraoperative or postoperative complications. Tacrolimus and mycophenolate mofetil were administered as the immunosuppression regimen. Warfarin was also used to prevent portal vein thrombosis, and the patient stopped taking lipid-lowering drugs after the operation. A significant decrease in the plasma level of TC (4.34 mmol/L) was observed one month after the operation (Fig. 2). Although there was initially a slight increase in the level of alanine aminotransferase (ALT), indicating liver damage, the index returned to the normal range within 6 months. He is currently taking medications including Tacrolimus and mycophenolate mofetil, with no liver transplant complications occurring during the 5-year follow-up. During the subsequent 5-year follow-up, lipid levels were re-examined regularly, with TC ranging from 3.79 to 5.33 mmol/L, and LDL-C from 2.37 to 3.82 mmol/L. The xanthomas also disappeared as the TC plasma level returned to normal (Fig. 3). The latest reexamination showed a TC of 4.95 mmol/L and an LDL-C of 3.10 mmol/L. Carotid ultrasound still reported IMT (left: 0.1 cm, right: 0.12 cm). Besides, the plaque on the anterior wall of the left common carotid artery trunk progressed (2.20 × 0.16 cm) and several plaques with a maximum size of approximately 1.71 × 0.17 cm (anterior wall of the common carotid artery trunk) can be seen on the inner wall of the right carotid artery. Echocardiography demonstrated both mild mitral and tricuspid regurgitation, and excess velocity (2.2 m/s) in the descending aortic arch.

## 3. Discussion

HoFH is a rare inherited disorder characterized by extremely high plasma levels of LDL-C since birth, accompanied by xanthomas and ASCVD. In the absence of treatment, HoFH patients often suffer from angiocardiopathy within the first two decades of life, and their survival depends on the level of plasma cholesterol before treatment [5]. Once diagnosed, the use of high-potency statins and ezetimibe with titration, along with lifestyle interventions, is commonly adopted as the first-line therapy. According to the Canadian Cardiovascular Society position paper, the lipid-lowering target is a 50% reduction in LDL-C, which should also fall below 2.5 mmol/L, or 2 mmol/L if ASCVD occurs [6]. In China, the LDL-C of pediatric patients should be <3.36 mmol/L, or reduced by at least 50%. Depending on the presence of ASCVD, the target value for adults is either 1.8 mmol/L or 2.6 mmol/L. The latest guideline from European Atherosclerosis Society pointed that in children and adolescents, an LDL-C goal of <3 mmol/L is recommended if treatment is initiated before 18 years and imaging assessment does not indicate ASCVD [7]. However, even with the maximum dose of statins and combination with other lipid-lowering drugs such as ezetimibe and bile acid chelating agents, almost no patients achieve the LDL-C targets.

The maximum dose of statins was not used in the currently report case due to intolerance. PSCK9 inhibitor was used and decreased the LDL-C level by approximately 30% in comparison to baseline, but this was still well above the target value. Despite the emergence of new drugs, LA is considered a pivotal treatment for the removal of lipoproteins from plasma, with proven efficacy and benefit for the prevention of cardiovascular events [8, 9]. Nevertheless, the application of LA is restricted by its high cost and the availability of local medical expertise. Furthermore, the weekly or biweekly treatment protocol reduces compliance. Currently, there is an increasing number of new targeted drugs that aim to lower LDL-C, such as PCSK9, lomitapide, mipomersen and evinacumab [10]. Consequently, liver transplantation appears to be the only option for curing HoFH, and is especially recommended for cases with severe myocardial disease.

Microsomal triglyceride transfer protein (MTTP) is an intracellular lipid-transfer protein expressed in hepatocytes and enterocytes. It participates in the formation of very low-density lipoprotein (VLDL) and chylomicrons (CM) by mediating the transfer of TGs to ApoB particles. Lomitapide, a selective inhibitor of MTTP, reduces plasma levels of LDL-C independently of LDLR by disrupting the assembly of ApoB-containing lipoproteins. Lomitapide was approved by the US Food and Drug Administration (FDA) as an adjuvant treatment for HoFH in 2012, and by the Committee for Human Medicinal Products (CHMP) in 2013 [11]. A single-arm, open-label, phase 3 study found that LDL-C was reduced by 50% from baseline after a 26-week combined treatment of lomitapide and traditional lipid-lowering medicine [12]. Adverse events included liver and gastrointestinal symptoms, as well as nausea and diarrhea. The long-term safety and efficacy of this treatment have been further elucidated [13, 14, 15, 16, 17, 18]. Whether the decrease in plasma LDL-C caused by lomitapide affects the incidence of subsequent cardiovascular events requires further study. HoFH is a rare disease and hence it is difficult to carry out large-scale research, which may also present ethical issues. A modeling analysis [19] carried out on the treatment of HoFH patients with lomitapide in combination with conventional agents has suggested there could be improved survival and a delayed incidence of major adverse cardiovascular events (MACEs). This treatment may also provide extra benefits when employed earlier. Nevertheless, the efficacy and safety of lomitapide for pediatric patients requires further study. Although lomitapide is not licensed for use in children, one report demonstrated promising effectiveness and manageable adverse events in HoFH patients [20].

Mipomersen is an antisense oligonucleotide that inhibits the production of ApoB-100 [21], which is the precursor of LDL, VLDL and apolipoprotein A (ApoA), thereby also decreasing plasma LDL-C. This drug exerts it function without being dependent on LDLR, and was therefore recommended as an adjunctive therapy for HoFH by the FDA in 2013. The use of mipomersen in HoFH patients in combination with the maximum tolerated dose of conventional lipid-lowering drugs resulted in an approximately 25% decrease in LDL-C from baseline in comparison with the placebo group [22]. Similar effects were also shown in patients with severe LDL hypercholesterolemia [23], familial hypercholesterolemia [24, 25], statin intolerance [26] and in pediatric HoFH patients [27]. As the level of plasma LDL-C is reduced, the incidence of cardiovascular events is also reduced [28], with the major adverse events being injection-site reactions, flu-like symptoms, and elevation of ALT. Hepatic steatosis is the most severe adverse event and is the mechanism-based consequence of mipomersen, thus putting in doubt its clinical application. The European Medicines Agency (EMA) refused the use of mipomersen based on safety concerns. However, a meta‑analysis of randomized clinical trials found that the use of mipomersen should not be stopped if it was effective and well tolerated [29]. Further studies on the long-term safety of mipomersen are warranted.

Angiopoietin-like 3 (ANGPTL3) regulates lipid metabolism by inhibiting lipoprotein lipase (LPL) and endothelial lipase (EL) [30]. Loss-of-function ANGPTL3 mutations are associated with reduced plasma TG, LDL-C and HDL-C levels in humans and are protective against coronary artery disease (CAD) [31]. Evinacumab is a full human monoclonal antibody inhibitor of ANGPTL3 that has a prominent role in the treatment of HoFH. In a double-blind, placebo-controlled phase 3 trial, intravenous infusion of evinacumab (15 mg/kg, every 4 weeks) or placebo was randomly applied in 65 patients with HoFH. The baseline level of LDL-C in the participants was 255.1 mg/dL, despite regular use of the maximum dose of lipid-lowering therapy, such as stains and ezetimibe. After 24 weeks of treatment, the plasma LDL-C level of patients receiving evinacumab treatment was reduced by 47.1% compared with the original level, while in the placebo group it increased by 1.9%. In addition, the plasma HDL-C level decreased by 30% in the treatment group. The most frequent adverse events were nasopharyngitis, influenza-like illness and headache. No patients discontinued the trial due to intolerance to adverse events. Two serious adverse events of urosepsis and a suicide attempt were reported in the evinacumab group, with the patients all recovering [32]. In light of the convenience for patients, a double-blind, placebo-controlled, phase 2 trial was conducted to evaluate the safety and efficiency of evinacumab with subcutaneous and intravenous administration [33]. The enrollment criteria were patients with refractory hypercholesterolemia, regardless of a background of HoFH. Efficacy and safety were estimated after 16 weeks. Subcutaneous evinacumab (450 mg, every week) was found to reduce LDL-C levels by 47.2% compared with baseline, thus demonstrating similar lipid-lowering effectiveness to intravenous evinacumab (15 mg/kg, every 4 weeks). Furthermore, there was no significant difference in the incidence of adverse events between subcutaneous evinacumab and intravenous evinacumab. Notably, an adverse event causing death was observed in a patient treated with subcutaneous evinacumab. Overall, these clinical trials demonstrate the potential application of evinacumab for the future treatment of HoHF. Whereas conventional lipid-lowering drugs like stains and PCSK9 inhibitors all rely on residual LDLR, evinacumab can lower the plasma lipid level while bypassing LDLR, thus providing options for HoFH patients with complete loss-of-function LDLR mutations [34]. However, more trials with larger sample sizes, longer follow-up times, and greater racial diversity are needed to further assess the long-term safety and effectiveness of evanicumab.

Since the majority of HoFH patients have mutations in LDLR, which is mainly expressed in the liver, liver transplantation seems to be the only way to cure refractory hypercholesterolemia in such patients. In 1984, the first liver transplantation combined with cardiac transplantation was performed in a 6-year-old girl with homozygous familial hypercholesterolemia and severe heart disease [4, 35]. Liver transplantation has since been adopted worldwide to cure HoFH [36, 37, 38, 39, 40, 41]. The plasma LDL-C level often dropped dramatically (up to 80%) within a month after liver transplantation, even going below the normal range with or without the use of lipid-lowering drugs. In addition, xanthoma faded away in parallel with the lowering of lipid levels. Angiographic evidence showing the regression of coronary artery disease after liver transplantation was also reported [42]. However, El-Rassi *et al*. [43] reported that a child died of sepsis 10 years after liver transplantation due to tight supra-valvular aortic narrowing and progressive severe bilateral coronary ostial stenosis, even though their lipid levels were always below the normal range. Due to the lack of an initial coronary artery condition before surgery, the most reasonable explanation might be that coronary artery disease existed before liver transplantation and progressed further even under normal lipid levels. A similar case was reported for a boy who had normal lipid levels after liver transplantation, but in which severe aortic valve stenosis still progressed [44]. These cases raise the question of the correct timing for liver transplantation in HoFH patients [45]. Liver transplantation was recommended after the development of cardiovascular disease for the consideration of immunological rejection, while further evidence found that liver transplantation may not reverse and could even aggravate serious coronary artery and valve disease after the normalization of lipid levels. Therefore, preemptive liver transplantation should be considered before the progression of cardiovascular disease [46]. Other issues also limit the application of liver transplantation to treat HoFH, including the availability of donor liver. In China, the donors for liver transplantation in children are mainly relatives. As an autosomal dominant disease, the parents of HoFH patients are inevitably heterozygous familial hypercholesterolemia cases with abnormal lipid metabolism, requiring the use of lipid-lowering drugs to lower their plasma levels to normal [47]. With the development of human induced pluripotent stem cells (hiPSCs) in 2006 [48], iPSC-derived livers may provide an additional option for HoFH patients in the future. The complications of liver transplantation cannot be ignored and include immunological rejection, hepatic artery thrombosis, and the improper use of immunosuppressants leading to aggravation of cardiovascular disease. This issue again raises the question of the optimal timing to conduct liver transplantation. The benefits and risks of liver transplantation must be carefully considered prior to the procedure. Finally, the long-term cardiovascular benefits of liver transplantation are unknown [49]. The follow-up time for most case reports to date have been short. Even if the plasma LDL-C level drops quickly after liver transplantation and xanthomas disappear, the long-term progression of plaques and valvular disease is relatively difficult to evaluate.

While this study provides the 5-year follow-up data for pediatric HoFH patients undergoing liver transplantation, three inherent limitations should be noted: Firstly, temporal constraint: the follow-up duration, though exceeding most comparable studies, remains inadequate to assess late graft-related complications (e.g., portal hypertension development), and puberty-associated lipid profile fluctuations. Besides, single-center design may limit generalizability to institutions with differing surgical protocol. Finally, sample size restriction reduces statistical power for rare adverse event detection. These factors notwithstanding, our medium-term findings establish crucial baseline data for future long-term investigations.

## 4. Conclusion

We report a case of liver transplantation for a child with HoFH in China and review the progress of treatment for this condition. Once diagnosed, lifestyle interventions and high-potency statins remain the first-line therapy for HoFH. The development of new drugs provides more choices for reaching target plasma lipid levels. Liver transplantation may still be the most effective treatment for HoFH in the long term. However, liver transplantation should be considered an effective treatment for HoFH rather than a curative option, highlighting the important clinical value of long-term lipid monitoring and cardiovascular risk assessment after surgery.

## Abbreviations

ALT, alanine aminotransferase; ANGPTL3, angiopoietin-like 3; ApoA, apolipoprotein A; ApoB, apolipoprotein B; ASCVDs, atherosclerotic cardiovascular diseases; CAD, coronary artery disease; CM, chylomicrons; EL, endothelial lipase; HDL-C, high-density lipoprotein cholesterol; hiPSCs, human induced pluripotent stem cells; HoFH, homozygous familial hypercholesterolemia; IMT, intimal thickness; LA, lipoprotein apheresis; LAD, left anterior descending; LDL-C, low-density lipoprotein cholesterol; LDLR, low-density lipoprotein receptor; LDLRAP1, low-density lipoprotein receptor adaptor protein 1; LPL, lipoprotein lipase; MACEs, major adverse cardiovascular events; MTTP, microsomal triglyceride transfer protein; PCSK9, proprotein convertase subtilisin kexin-9; TC, total cholesterol; TGs, triglycerides; VLDL, very low-density lipoprotein.
